# The sinus venosus myocardium contributes to the atrioventricular canal: potential role during atrioventricular node development?

**DOI:** 10.1111/jcmm.12525

**Published:** 2015-03-06

**Authors:** Tim P Kelder, Rebecca Vicente-Steijn, Tom J Harryvan, Georgios Kosmidis, Adriana C Gittenberger-de Groot, Rob E Poelmann, Martin J Schalij, Marco C DeRuiter, Monique RM Jongbloed

**Affiliations:** a Department of Anatomy & Embryology, Leiden University Medical CenterLeiden, The Netherlands; b Department of Cardiology, Leiden University Medical CenterLeiden, The Netherlands

**Keywords:** cardiac conduction system development, lineage tracing, sinus venosus myocardium, atrioventricular canal, atrioventricular node, atrioventricular nodal reentrant tachycardia

## Abstract

The presence of distinct electrophysiological pathways within the atrioventricular node (AVN) is a prerequisite for atrioventricular nodal reentrant tachycardia to occur. In this study, the different cell contributions that may account for the anatomical and functional heterogeneity of the AVN were investigated. To study the temporal development of the AVN, the expression pattern of ISL1, expressed in cardiac progenitor cells, was studied in sequential stages performing co-staining with myocardial markers (TNNI2 and NKX2-5) and HCN4 (cardiac conduction system marker). An ISL1+/TNNI2+/HCN4+ continuity between the myocardium of the sinus venosus and atrioventricular canal was identified in the region of the putative AVN, which showed a pacemaker-like phenotype based on single cell patch-clamp experiments. Furthermore, qPCR analysis showed that even during early development, different cell populations can be identified in the region of the putative AVN. Fate mapping was performed by *in ovo* vital dye microinjection. Embryos were harvested and analysed 24 and 48 hrs post-injection. These experiments showed incorporation of sinus venosus myocardium in the posterior region of the atrioventricular canal. The myocardium of the sinus venosus contributes to the atrioventricular canal. It is postulated that the myocardium of the sinus venosus contributes to nodal extensions or transitional cells of the AVN since these cells are located in the posterior region of the AVN. This finding may help to understand the origin of atrioventricular nodal reentrant tachycardia.

## Introduction

The cardiac conduction system (CCS) initiates electrical activation of the heart and ensures proper electrical propagation resulting in coordinated mechanical activation of the myocardium. Disturbances in the normal electrical activation pattern of the heart resulting in cardiac arrhythmias are an important cause of mortality and morbidity in the general population 1.

During early cardiac development fusion of the bilateral cardiogenic plates establishes the primary heart tube (PHT). During further development a subpopulation of cells from the splanchnic mesoderm continues to differentiate towards a cardiac fate and is recruited to the PHT at the venous and arterial poles. The splanchnic mesoderm at the venous pole gives rise to the myocardium of the sinus venosus. This myocardium surrounds the cardinal and pulmonary veins and shows distinct expression patterns as compared with the atrial working myocardium 2. In contrast to the atrial myocardium, the sinus venosus myocardium expresses *Tbx18* 3, ISL1 2,4, *Shox2* 5, *Pdgf-receptor alpha* 6, *Tbx3* 7,8 and RHOA 2, while it is negative for NKX2-5 during early stages of development 3,9. The sinus venosus myocardium includes the definitive right-sided sinoatrial node (SAN) as well as a transient left-sided SAN, and will differentiate into a working myocardial phenotype during further development, with exception of the future right-sided SAN. The SAN will retain a primitive phenotype as characterized by relatively high automaticity, slow conduction and poor coupling of cells and becomes the dominant pacemaker of the heart 10. The right and left venous valves are also components of the sinus venosus myocardium and are known substrates for atrial fibrillation and tachycardia in the fully developed heart 1. They extend along the posterior wall of the right atrium towards another important structure of the CCS, the atrioventricular node (AVN). The AVN delays conduction of the electrical impulse generated by the SAN, thereby ensuring adequate filling of the ventricles. The AVN is part of the myocardium of the atrioventricular (AV) canal and consists of a compact node, situated in the posterior part of the AV canal, covered by transitional cells as well as nodal extensions running towards the vestibules of the mitral and tricuspid valves 11. The exact origin of the individual components of the AVN is still unclear. Studies indicate that at least the compact node derives from the myocardium of the AV canal 12,13, suggesting that the majority of cells of the AVN is formed from one source of cells. However, the presence of electrophysiologically and morphologically distinct cells (*i.e*. compact nodal cells, transitional cells and nodal extensions) in the region of the AVN indicates contributions from different sources, and/or with different transcriptional programmes. A dual contribution would be a possible explanation for the occurrence of these different cell types and may help to understand the occurrence of atrioventricular nodal reentrant tachycardia (AVNRT). A prerequisite for AVNRT to occur is the presence of electrophysiologically distinct pathways within the AVN 14, resulting in slow conduction in the so-called slow pathway, unidirectional conduction in the fast pathways and a central area of block. The slow pathway is a common target for catheter ablation of AVNRT, and has been described anatomically as the inferior nodal extension 15. It has been postulated that the transitional cells and nodal extensions are an atrial contribution to the AVN 16 or are derived from the myocardium of the sinus venosus 2. Interestingly, the inferior nodal extension has a similar gene expression profile as the SAN 17, and pacemaker activity from the AV junction has been attributed to the inferior nodal extension 18, thereby suggesting a developmental relation between the AVN and the sinus venosus myocardium.

ISL1, a LIM homeodomain transcription factor that plays an important role during cell proliferation, differentiation and survival 19, is expressed in the sinus venosus myocardium during early stages of development. Genetic lineage tracing with an inducible *Isl1-Cre* revealed important differences in timing of addition of cells to the developing heart and AVN. When *Cre-*recombinase was induced with Tamoxifen at embryonic day (E)7, a large number of *Isl1*-positive cells were found in the heart at E9. Induction of *Cre* at E9 and analysis at E11 revealed far less *Isl1*-positive cells, with a cluster of cells in the region of the developing AVN 20. This could indicate that the AVN develops from multiple sources, with contributions at different time-points during development. Immunohistochemical analysis of ISL1 expression in chick embryos revealed a region of ISL1+ cells in the dorsal atrial wall, just cranial to the AV junction, which is known to be the location of the future AVN, leading to the hypothesis of a contribution of the sinus venosus myocardium to the AVN 2. However, no cell lineage tracing studies independent of gene expression to prove a contribution from the sinus venosus myocardium have been performed.

In this study, vital dye labelling experiments aimed at studying different contributions to the developing AVN were performed, to determine the origin of cells contributing to the AVN. First, expression patterns marked by double or triple staining with ISL1 (expressed in cardiac progenitor cells), the myocardial markers TNNI2 and NKX2-5 and the CCS marker HCN4, were studied in sequential stages of chick and mouse development. These results were used as a roadmap for the targeted vital dye labelling experiments.

## Materials and methods

### Immunohistochemistry

To obtain a developmental series of chicken heart development, fertilized eggs of the White Leghorn chicken were incubated at 37°C and 80% humidity. Embryos (HH7-31) were excised, staged according to Hamburger and Hamilton criteria 21, and fixed in 4% paraformaldehyde for 24 hrs. Subsequently, they were embedded in paraffin and sectioned serially (5 μm) for immunohistochemical analysis.

To confirm the results obtained in chick embryos, immunohistochemical analysis was extended to wild-type mouse embryos with a mixed genetic background of E10.5, E11.5 and E12.5 (mouse line has been described previously 22). The morning of the vaginal plug was considered E0.5. Pregnant dams were euthanized with CO_2_ exposure followed by cervical dislocation. Processing of embryos after excision was similar to the chicken embryos. Animal care was in accordance with national and institutional guidelines and approved by the animal experiments committee of the Leiden University Medical Center (Permit Number 13067).

Serial sections were rehydrated and subjected to microwave antigen retrieval in citric acid buffer (pH = 6.0) before staining with the primary antibodies. Sections were incubated with primary antibodies against ISL1 (Clone 40.2d6, 1/100; Developmental Studies Hybridoma Bank, Iowa City, IA, USA), Troponin I, isoform 2 (TNNI2) (SC-15368, 1/200; Santa Cruz Biotechnology Inc., Dallas, TX, USA), NKX2-5 (SC-8697, 1/500; Santa Cruz Biotechnology Inc.) and HCN4 (APC-052, 1/2000; Alomone Labs, Jerusalem, Israel) overnight. Primary antibodies were diluted in PBS-Tween-20 with 1% bovine serum albumin (BSA, A8022; Sigma-Aldrich, St. Louis, MO, USA) to prevent non-specific binding. Between subsequent incubation steps all slides were rinsed in PBS (2×) and PBS-Tween-20 (1×). To enhance the signal of ISL1, horse anti-mouse-Biotin (BA-2000, 1/200; Vector Laboratories Inc., Burlingame, CA, USA) in PBS-Tween-20 was added together with normal horse serum (6-S-2000, 1/66; Brunschwig Chemie, Switzerland) for 60 min. Visualization of the primary antibodies was achieved by incubation with fluorescently labelled secondary antibodies, diluted in PBS-Tween-20 (60 min.). The following antibodies were used: Alexa Fluor® 488 Streptavidin Conjugate (S-11223, 1/200), Alexa Fluor® 555 donkey anti-rabbit IgG (A-31572, 1/200) and Alexa Fluor® 647 donkey anti-goat IgG (A-21447, 1/200). All secondary antibodies were purchased from Life Technologies (Carlsbad, CA, USA). DAPI (D3571, 1/1000; Life Technologies) was used as a nuclear stain and the slides were mounted with Prolong gold (Life Technologies). To reduce interfering autofluorescence (HH7-11), small adjustments were made to the above described protocol. Sections were blocked (30 min.) with a commercially available blocking reagent (cat. no. 11 096 176 001; Roche, Mannheim, Germany), which was also used to dilute the primary and secondary antibodies. The sections were incubated with the primary antibody for 2 hrs. Sections were visualized using a Leica DM 5500 fluorescence microscope (Leica Microsystems, Buffalo Grove IL, USA). ImageJ was used to process the pictures.

### *In situ* hybridizations

For detection of *HCN4* mRNA expression in the developing CCS, *in situ* hybridizations in chick embryos were performed (HH15-23) and analysed as described previously 23.

### Laser capture microdissection procedure

Laser capture microdissection (LCM) was performed to specifically isolate tissue of three distinct structures: 1. Myocardial continuity sinus venosus and AV canal (SV-AVC); 2. More caudal region of posterior AV canal (AVC); 3 Lateral wall right ventricle (RV). Embryos of HH21 were extracted and fixated for 24 hrs in 4% paraformaldehyde, embedded in paraffin and serially sectioned at 5 μm. Sections were mounted on membrane-covered slides (MembraneSlide 1.0 PEN; Carl Zeiss Microscopy, Thornwood, NY, USA). Tissue from the different structures of interest were dissected using the PALM microbeam (Carl Zeiss Microscopy) and RoboSoftware package and subsequently transferred to AdhesiveCaps (Carl Zeiss Microscopy). After microdissection, the tissue was stored at −80°C. The outline of the dissected tissue was photographed in all embryos and examples of the different regions before and after dissection are shown in Figure S1.

### RNA isolation, cDNA synthesis and qPCR of laser microdissection tissue

After microdissection, RNA was isolated using the RecoverAll™ Total Nucleic Acid Isolation Kit for FFPE (Ambion, Life Technologies). cDNA synthesis was performed with the iScript™ cDNA synthesis kit (Bio-Rad, Hercules, CA, USA). After cDNA synthesis, qPCR was performed for *TNNT2, NKX2-5, HCN4* and *ISL1*. For *ISL1* a nested approach was necessary to detect mRNA expression. After cDNA synthesis, for *ISL1*, 15 cycles of pre-amplification were performed with 10 μl SybrGreen Mastermix (Bio-Rad), 8.5 μl nuclease free water, 0.25 μl forward and 0.25 μl reverse primer and 1 μl of cDNA (final volume of 20 μl). For the other three genes, no pre-amplification was needed. The qPCR was performed with 10 μl of SybrGreen Mastermix (Bio-Rad), 1 μl of forward primer, 1 μl of reverse primer, 1 μl of cDNA (or 1 μl nuclease free water as negative control) and 7 μl nuclease free water per reaction (final volume 20 μl). Reactions were carried out in triplicate for each sample. qPCR was performed on a Bio-Rad CFX96 real-time system. Melting curve analysis was performed to verify single PCR product amplification. Chicken *GAPDH* was used as the reference standard for normalization, and relative differences in mRNA expression were determined. The primers used are described in Table1.

**Table 1:** qPCR primers

Primer	Sequence
*GAPDH forward*	5′-CTAAGGCTGTGGGGAAGGT-3′
*GAPDH reverse*	5′-GTTGTTGACCTGACCTGCC-3′
*TNNT2 forward*	5′-AAGAAGGGTGGCAAGAAGCA-3′
*TNNT2 reverse*	5′-CTCGTGGTCACTGACACGATTT-3′
*NKX2-5 forward*	5′-CCCTACTACGTGAAGAGCTACG-3′
*NKX2-5 reverse*	5′-TCGGGATCCTCCAGCTCTCT-3′
*HCN4 forward*	5′-GCCTTCTGCTGTTGGCTCT-3′
*HCN4 reverse*	5′-GCTGCTGGATGTGGTAGGA-3′
*ISL1 forward*	5′-AAAAGAAGCATTATGATGAAGCAA-3′
*ISL1 reverse*	5′-CATGTCTCTCCGGACTAGCAG-3′
*ISL1 nested forward*	5′-AGCAACCCAATGACAAAAC-3′
*ISL1 nested reverse*	5′-TGTCTCTCCGGACTAGCAG-3′

### Whole mount ISL1 staining

HH21 embryos were extracted and the heart was carefully removed from the thorax under the preparation microscope. The heart was fixated in DMSO:100% Methanol (ratio 4:1) for 2 hrs at 4°C, after which the tissue was rehydrated in 75%, 50% and 25% methanol in PBS. After rinsing thoroughly with PBS for 30 min. at room temperature, the tissue was permeabilized with PBS-Tween for 1 hr. Blocking was performed with PBS-Tween-20 with 1% BSA for 1 hr at room temperature. The ISL1 primary antibody (Clone 40.2d6, 1/100; Developmental Studies Hybridoma Bank), was dissolved in PBS-Tween-20 with 1% BSA and the tissue was incubated with the antibody overnight at 4°C in a shaking plate. The next morning, the tissue was rinsed with PBS and incubation with Vectastain ABC staining kit (PK- 6100; Vector Labs) was performed for 90 min. at room temperature. After rinsing with PBS, the tissue was incubated for 30 min. in 3-30diaminobenzidinetetrahydrochloride (DAB) (D5637; Sigma-Aldrich) dissolved in Tris/Maleate (pH 7.6). Finally, a fresh solution of DAB with 5 μl of H_2_O_2_ was added for a brief moment (±5 sec.) to the tissue, after which the tissue was thoroughly rinsed in PBS to stop the visualization reaction.

### Single cell patch-clamp recordings

Electrophysiological data were obtained by single cell patch-clamp of HH21-22 chick embryonic cardiomyocytes of the continuity between the sinus venosus and AV canal and compared to cardiomyocytes from the RV. Whole mount ISL1 stained embryos were used to identify the ISL1+ continuity between the sinus venosus and AV canal, which aided in specifically dissecting this tissue under the preparation microscope. As control tissue, a piece of RV was collected. The dissected tissue was collected in 1.5 ml tubes and 800 μl of 0.05% Trypsin (Life Technologies) was added. The tube was placed in a heated (37°C), shaking plate for 10 min. After this, the tubes were vortexed until all tissue was dissociated and centrifuged at 114 g for 5 min. The supernatant was removed and the cells were resuspended in medium containing DMEM high glucose (Life Technologies), 10% foetal calf serum (Sigma-Aldrich), 1% penicillin-streptomycin (Life Technologies), 1% non-essential amino acids (Life Technologies) and 1% L-Glutamine (Life Technologies). Cells were plated on glass coverslips and used for single cell patch-clamp recording.

Recordings were performed on single cardiomyocytes, 1–2 days after cell dissociation. Action potential measurements of spontaneously contracting cells were recorded. Action potentials were recorded with the perforated patch-clamp technique using an Axopatch 200B amplifier (Molecular Devices Sunnyvale, CA, USA) and were filtered (5 kHz) and digitized (40 kHz). Data were acquired with pClamp10.1 (Molecular Devices, Sunnyvale, CA, USA) and analysis was performed with custom-made software. Potentials were corrected for the estimated change in liquid junction potential.

Action potentials were recorded at 37°C in Tyrode's solution. The pipette solution contained (mM): K-gluconate 125, KCl 20, NaCl 5, amphotericin-B 0.22, HEPES 10; pH 7.2 (KOH). The maximal diastolic potential (MDP), maximal upstroke velocity (V_max_), action potential amplitude, and action potential duration (APD) at 50% and 90% repolarization (APD_50_ and APD_90_, respectively) were analysed. Data from 8 to 9 consecutive action potentials were averaged.

### Micro-injection procedure

A small window was created in the shell to gain access to the embryo (HH15-17). A 1:1 solution of two fluorescent dyes (DiI and 5-TAMRA) was used to label and follow cells during development. 2.5 mg DiI (D-282; Life Technologies) was dissolved in 50 μl DMSO, which in turn was diluted in 950 μl ethanol (100%). 2.5 mg 5-TAMRA (C-2211; Life Technologies) was dissolved in 1000 μl DMSO. The combined solution was loaded into a pulled glass needle and injected in different regions, using a programmable microinjector (IM-300 Narishige, Tokyo, Japan) and micromanipulator.

**Figure 1:**
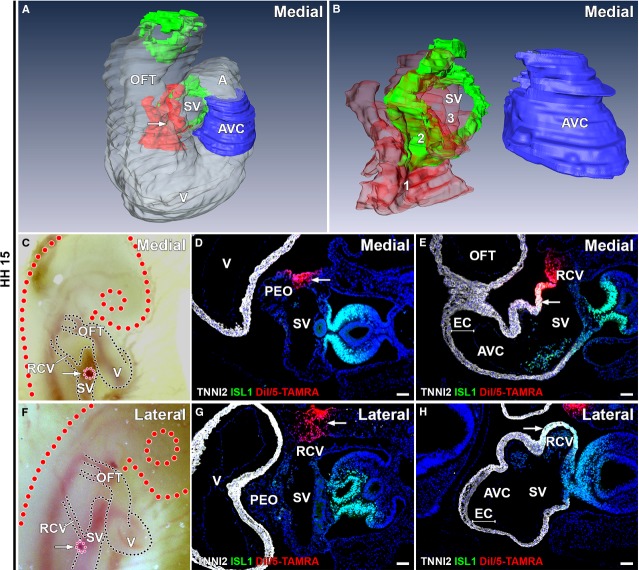
Medial and lateral labelling at HH15. (A–B) 3D reconstructions of medial labelling, white: myocardium, blue: AV canal myocardium, green: ISL1+/TNNI2+ sinus venosus myocardium, red: DiI/5-TAMRA labelling. (A) Overview of PHT, arrow indicates labelling. (B) All 14 microinjections are superimposed to clarify extend of total labelling. For separate labels, see interactive PDF (Data S1). No labelling of AV canal myocardium is seen and distance between myocardium of sinus venosus and AV canal is present. Labelling is found in mesenchyme RCV (1 in B, 100% of embryos), ISL1+/TNNI2+ sinus venosus myocardium (2 in B, 100% of embryos) and a small region of ISL1-/TNNI2+ myocardium directly bordering the ISL1+/TNNI2+ sinus venosus (3 in B, 64% of embryos). (C and F) Directly after labelling demonstrates *in ovo* medial (C) and lateral (F) location of DiI/5-TAMRA (pink, arrow). (D and G) DiI/5-TAMRA in mesenchyme RCV present in medial (D) and lateral (G) labelling. (E and H) DiI/5-TAMRA present in ISL1+/TNNI2+ sinus venosus myocardium in medial labelling (DiI/5-TAMRA: arrow in E), not in lateral labelling (corresponding DiI/5-TAMRA negative region: arrow in H). AVC: atrioventricular canal; EC: endocardial cushion; OFT: out-flow tract; PEO: proepicardial organ; RCV: right cardinal vein; SV: sinus venosus; V: ventricle. (D, E, G and H) White: TNNI2, green: ISL1, red: DiI/5-TAMRA, blue: DAPI; scale bars: 50 μm.

Medial labelling was performed by opening the coelomic cavity with subsequent injection in the ISL1+/TNNI2+ sinus venosus myocardium (Fig.1A–E). To calculate the volume of labelling, all microscopic sections that contained DiI/5-TAMRA were photographed and ImageJ was used to calculate the volume of labelling. The total volume of labelling and the volume of labelling in the ISL1-/TNNI2+ atrial myocardium directly bordering the ISL1+/TNNI2+ sinus venosus myocardium were calculated for the 14 embryos that were analysed in the medial group 1–2 hrs after labelling.

Lateral, right-sided labelling of the mesenchyme and vessel wall of the right cardinal vein (RCV) was performed just caudal to the entrance of the RCV in the sinus venosus (Fig.1F–H). Injection in this region was performed through the developing lateral body wall leaving the coelomic cavity intact. This injection site was chosen to assure lateral labelling of mesenchymal structures and to minimize leakage to other cardiac components. Embryos were collected at selected time-points (HH15-25) to assess the location of labelling.

### 3D reconstruction

AMIRA (Template Graphics Software, Inc. Houston, TX, USA) reconstructions aided in clarifying the three-dimensional relationship between different structures. Embryos were serially sectioned and immunofluorescently stained for both TNNI2 and ISL1. The slides were photographed and processed with Adobe Photoshop CS6. Finally, the slides were stacked and aligned and structures of interest were labelled with the AMIRA software. A subset of reconstructions was converted to 3D interactive PDFs, which are available online. Different functions (*e.g*. buttons to show, hide or make different structures transparent) were included in the interactive PDFs, using Javascript. The scripts that were used were previously written and published 24 and slightly modified for the current PDFs.

### Statistical analysis

Results are expressed as mean ± SEM. Comparisons were made using unpaired Student's *t*-test (normal distribution) or Mann–Whitney *U*-test when data were not normally distributed. *P* < 0.05 was considered statistically significant.

## Results

### Identification of an ISL1+/TNNI2+/HCN4+ myocardial continuity between the sinus venosus myocardium and posterior portion of the AV canal

**Figure 2:**
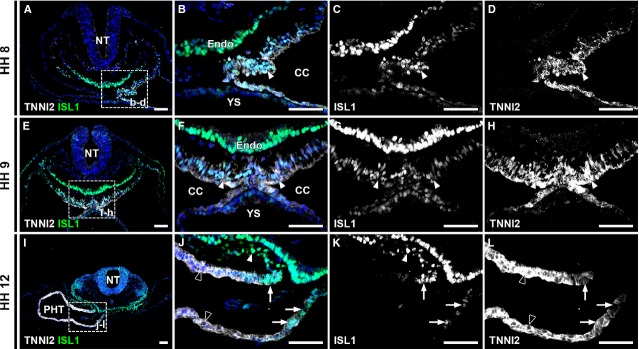
ISL1 and TNNI2 co-expression at HH stage 8-12. (A) Overview of an HH8 embryo includes foregut and left sided cardiogenic plate. The boxed region is enlarged in (B–D). (B) co-expression of TNNI2 and ISL1 in cardiogenic plate (arrowheads in B–D). (C–D) Separate channels in grey values. (E) Overview of an HH9 embryo, with boxed region enlarged in (F–H) Co-expression of ISL1 and TNNI2 in majority of cells in cardiogenic plate (arrowheads in F–H). (I) Overview of an HH12 embryo. Boxed region is shown at higher magnification in J–L. (J) ISL1+/TNNI2+ myocardium (arrows) is shown at arterial pole of primary heart tube. White arrowheads show ISL1+/TNNI2- splanchnic mesoderm. Note lower expression of TNNI2 in ISL1+ cells. Black arrowheads show ISL1-/TNNI2+ myocardium of primary heart tube. (K) ISL1 expression is shown. Arrowheads show ISL1+/TNNI2- splanchnic mesoderm and arrows show ISL1+/TNNI2+ myocardium of arterial pole. (L) TNNI2 expression at arterial pole. Arrows show decreased TNNI2 expression in ISL1+ cells. CC: coelomic cavity; Endo: endoderm; NT: neural tube; PHT: primary heart tube; YS: yolk sac. White: TNNI2, green: ISL1, blue: DAPI; scale bars: 50 μm.

This first phase of the study was aimed at analysis of expression patterns of ISL1, performing immunostaining at stages HH7-31. The first expression of TNNI2 was seen by HH8, within the medial borders of the bilateral cardiogenic plates, co-localizing with ISL1 (Fig.2A–D). Strong expression of ISL1 was also found in the foregut endoderm (Fig.2A–C). At HH9, the bilateral plates of mesoderm met in the midline, and started to fuse (Fig.2F). TNNI2 expression increased and co-localization with ISL1 was still found in the majority of cells (Fig.2E–H). By HH10, the bilateral plates had fused and formed a semitubular structure, with the ventral side formed by the TNNI2+ myocardium, and the dorsal side bordered by the ISL1+ foregut endoderm. By HH11-12, the heart is almost closed posteriorly, forming the PHT. Most cardiomyocytes of the tube have lost ISL1 expression (Fig.2I and J), except for populations of ISL1+/TNNI2+ cells at the arterial (Fig.2I–L) and venous pole.

**Figure 3:**
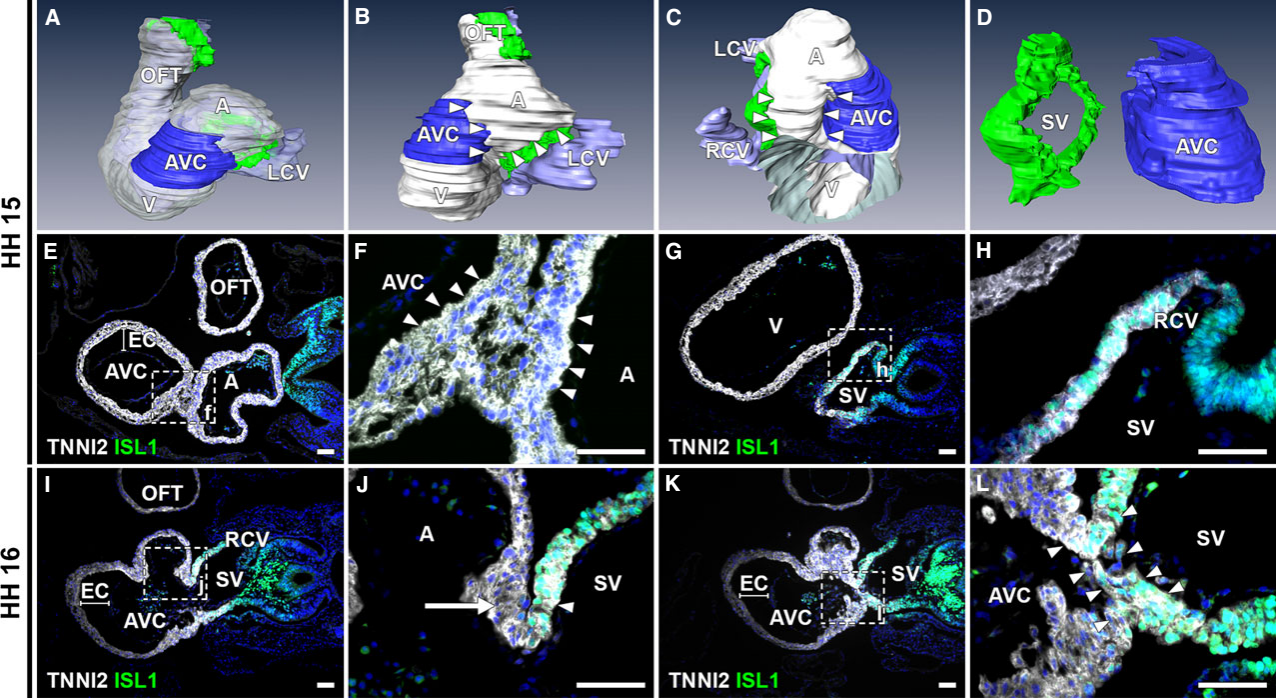
ISL1 and TNNI2 expression at the venous pole at HH15-16. (A–D) 3D reconstructions, HH15 embryo. White: myocardium, blue: AV canal myocardium, green: ISL1+/TNNI2+ sinus venosus myocardium, purple: lumen (B). Left lateral view showing ISL1-/TNNI2+ myocardium (between arrowheads) between myocardium of AV canal and sinus venosus. (C and D) Right lateral view with (in C, part of ventricle and entire OFT are removed to expose right portion of inflow tract) and without (in D) myocardium reconstructed, demonstrating distance between sinus venosus and AV canal myocardium (between arrowheads). (E and F) Transverse section at HH15 of ISL1-/TNNI2+ myocardial connection between AV canal (identified based on morphological criteria, *i.e*. presence of thick endocardial cushions and underlying spongious myocardium) and lower region of atrium (between arrowheads). (G and H) ISL1+/TNNI2+ sinus venosus (including RCV myocardium), which is located caudally to connection between atrium and AV canal. (I and J) At HH16, RVV is a double leaflet with ISL1+/TNNI2+ myocardium in inner leaflet (arrowhead) and ISL1-/TNNI2+ myocardium in outer leaflet (arrow). (K and L) ISL1+/TNNI2+ continuity between the SV and AV canal (between arrowheads) is first seen at HH16. A: atrium; AVC: atrioventricular canal; EC: endocardial cushion; LCV: left cardinal vein; OFT: out-flow tract; RA: right atrium; RCV: right cardinal vein; SV: sinus venosus. White: TNNI2, green: ISL1, red: NKX2-5 blue: DAPI; scale bars: 50 μm.

Analysis of ISL1 expression in subsequent developmental stages revealed ISL1/TNNI2 co-expression in the myocardium of the sinus venosus. At HH14-15, the myocardium connecting the atria to the AV canal is negative for ISL1 (Fig.3A–F). The AV canal was defined based on morphological criteria, *i.e*. the presence of thick endocardial cushions and less densely organized myocardium underlying the cushions (Fig.3E, I and K). More caudally the sinus venosus myocardium is positive for ISL1 and TNNI2 (Fig.3G and H). Thus, there is initially a region of ISL1-/TNNI2+ myocardium between the sinus venosus myocardium and the AV canal myocardium (Fig.3B and C). To further clarify the relation between the different structures, an interactive PDF of the three-dimensional reconstruction shown in Figure3 is available online (Data S1). At this stage, the RVV and LVV are not evident, yet.

During further development, the RVV becomes recognizable as a double myocardial leaflet, of which the inner leaflet (SV luminal side) shows co-expression of ISL1 and TNNI2. The outer leaflet is ISL1-/TNNI2+ (atrial luminal side; Fig.3I and J). An ISL1+/TNNI2+ continuity between the myocardium of the sinus venosus and the posterior part of the AV canal becomes apparent at HH16-17 (Fig.3K and L).

**Figure 4:**
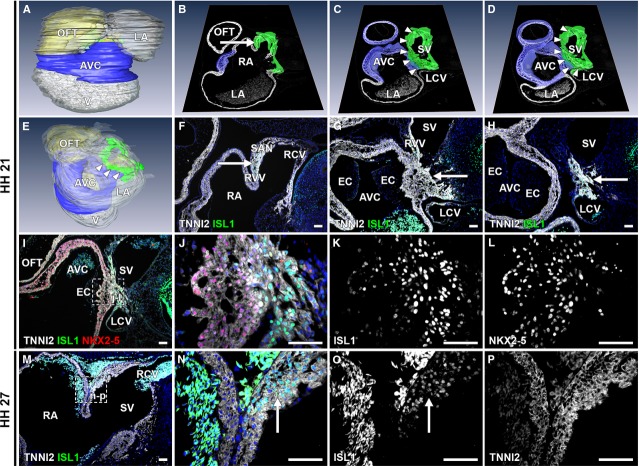
description of ISL1+/TNNI2+ continuity between myocardium of sinus venosus and AV canal. (A–E) 3D reconstructions, HH21 embryo. White: myocardium, yellow: mesenchyme, blue: AV canal myocardium, green: ISL1+/TNNI2+ sinus venosus myocardium. (A) Overview of heart. (E) Arrowheads indicate continuity between sinus venosus and AV canal myocardium. (B–D) 3D reconstructions of AV canal and sinus venosus myocardium with in white TNNI2 section from AMIRA. (B–D) Cranial to caudal. (F–H) Corresponding TNNI2/ISL1 staining. (B) Location of SAN and RVV (arrow). (F) ISL1+/TNNI2+ connection between SAN and RVV (arrow). (C) SAN and RVV are attached to myocardial continuity between sinus venosus and AV canal (arrowheads), arrow in G. AV canal identified by presence of endocardial cushions and spongious myocardium. (D) Connection between LCV and ISL1+/TNNI2+ sinus venosus. (I–L) ISL1/TNNI2/NKX2-5 staining of myocardial continuity between sinus venosus and AV canal. (K) decrease in ISL1 expression from sinus venosus towards AV canal. (L) opposite expression pattern for NKX2-5. (M–P) ISL1 expression in SAN (arrows) at HH27. (O and P) Grey values for ISL1 and TNNI2, showing ISL1+/TNNI2+ SAN. AVC: atrioventricular canal; EC: endocardial cushion; LA: left atrium; LCV: left cardinal vein; OFT: outflow tract; RA: right atrium; RCV: right cardinal vein; SV: sinus venosus. (F–P) White: TNNI2, green: ISL1, red: NKX2-5, blue: DAPI; scale bars: 50 μm.

At HH 21, this continuity still shows ISL1 and TNNI2 expression and is located between the posterior portion of the AV canal (region of the (putative) AVN) and the cranial portion of the sinus venosus at the level of the entrance of the cardinal veins in the sinus venosus (Fig.4E). The AV canal is again characterized by the presence of thick endocardial cushions with spongious myocardium underlying the cushions (Fig.4G–J). The ISL1+/TNNI2+ area includes the myocardium of the SAN, RCV, RVV and LCV, including the left SAN (Fig.4A–H). The central part of the sinus venosus myocardium is connected to the myocardium of the posterior AV canal (Fig.4C, E and G), where ISL1 expression is seen in the AV canal myocardium (Fig.4J and K). An interactive PDF of the three-dimensional reconstruction is available online, which shows in further detail the continuity between the ISL1+/TNNI2+ sinus venosus myocardium and the posterior AV canal (Data S2). An ISL1/NKX2-5/TNNI2 triple staining was performed and showed a gradient in ISL1 expression from the SV myocardium (strongest expression) towards the AV canal myocardium (decrease in expression) (Fig.4I–L). NKX2-5 expression showed a similar but opposite gradient (Fig.4I–L), suggesting differentiation of ISL1+/TNNI2+/NKX2-5- sinus venosus cardiomyocytes towards NKX2-5+/TNNI2+/ISL1- AV canal cardiomyocytes.

During further development, ISL1 expression became confined to the myocardial sleeve of the RCV at the location of the future SAN (Fig.4M–P), with no discernible expression of ISL1 in the continuity between the myocardium of the sinus venosus and AV canal any more.

**Figure 5:**
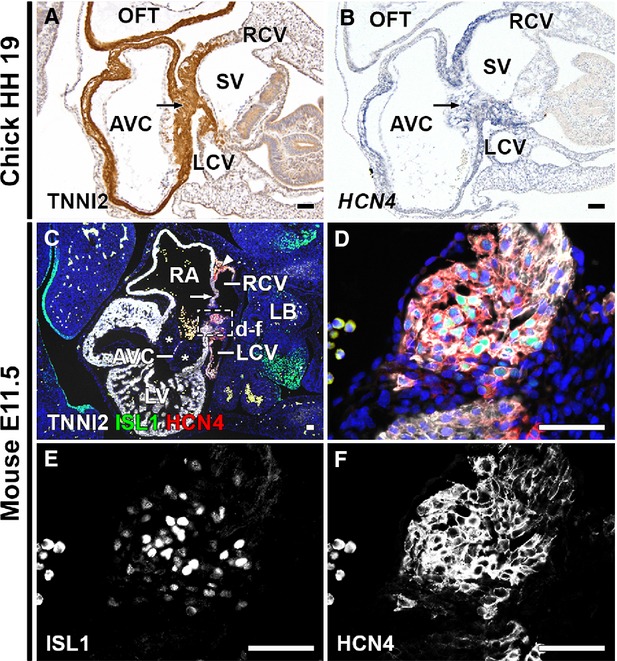
HCN4 expression in myocardial continuity between sinus venosus and posterior AV canal. (A) TNNI2 stain of HH19 chick embryo. Arrow indicates continuity between sinus venosus myocardium and posterior AV canal. (B) ISH shows *HCN4*mRNA expression in continuity (arrow). (C) Merge of ISL1, TNNI2, HCN4 and DAPI staining of E11.5 mouse embryo, showing the myocardial continuity between the sinus venosus and posterior AV canal. The ISL1+/HCN4+/TNNI2+ SAN (arrowhead) and RVV (arrow) are shown. Note the thick endocardial cushions (asterisks) in the AVC. (D–F) Higher magnifications of boxed area in C and D. ISL1+/TNNI2+/HCN4+ cells are seen in the continuity between sinus venosus myocardium and posterior AV canal myocardium. (E and F) Grey values of ISL1 (E) and HCN4 (F). AVC: atrioventricular canal; LA: left atrium; LB: long bud; LCV: left cardinal vein; LV: left ventricle; OFT: outflow tract; RA: right atrium; RCV: right cardinal vein; SV: sinus venosus; V: ventricle. (C–F) White: TNNI2, green: ISL1, red: HCN4, blue: DAPI; scale bars: 50 μm.

To investigate whether the myocardial continuity between the sinus venosus and posterior AV canal indeed had a ‘pacemaker-like’ phenotype, *in situ* hybridizations were performed in chick embryos for *HCN4*, which is used as a marker for the (developing) CCS 23. The connection between the sinus venosus myocardium and the myocardium of the posterior portion of the AV canal (Fig.5A) showed *HCN4* expression (Fig.5B), shown at HH19. To verify the co-localization of ISL1, TNNI2 and HCN4 in this region in mammals, a triple staining for ISL1, TNNI2 and HCN4 was performed in wild-type mouse embryos. ISL1+/TNNI2+/HCN4+ cells were found in the region of the myocardial continuity between the sinus venosus and AV canal (shown at E11.5 in Fig.5C–F). These results indicate that the cells of the myocardial continuity between the sinus venosus and posterior AV canal have a pacemaker-like phenotype.

### Characterization of the myocardial continuity between the sinus venosus and AV canal confirms the pacemaker-like phenotype

**Figure 6:**
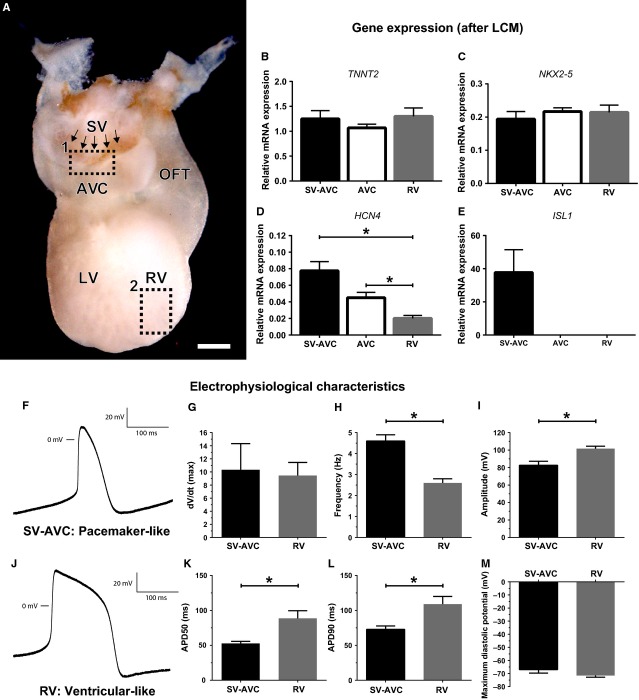
Characterization of the myocardial continuity between the sinus venosus and AV canal. (A) Whole mount ISL1 staining, posterior view of the heart. Arrows indicate ISL1+ cells in continuity sinus venosus and AV canal. Rectangle 1 shows region from which SV-AVC continuity cells were collected. Rectangle 2 shows origin of right ventricular cells for patch clamp. (B–E) mRNA expression after laser capture microscopy (LCM) of *TNNT2, NKX2-5, HCN4* and *ISL1*. (F–M) Results of single cell patch-clamp experiments. (F and J) Representative tracing of SV-AVC continuity cells (F, pacemaker-like tracing) and RV cells (J, ventricular-like phenotype). (G–I, K–M) Graphs showing the difference in maximum upstroke velocity (G), frequency (H), amplitude (I), APD50 (K), APD90 (L) and maximum diastolic potential (M). * Indicates *P* < 0.05. AVC: AV canal; LCM: laser capture microscopy; LV: left ventricle; OFT: outflow tract; RV: right ventricle; SV: sinus venosus; SV-AVC: myocardial continuity sinus venosus and AV canal; scale bar: 250 μm.

Laser capture microdissection was performed to characterize the sinus venosus-AV canal continuity (SV-AVC in Fig.6) and to quantify difference in expression levels of *TNNT2, NKX2-5, ISL1* and *HCN4* in this region. These results were compared to the more caudal tissue of the posterior AV canal and tissue from the lateral wall of the RV to better identify the phenotype of the SV-AVC region.

Expression of *TNNT2* and *NKX2-5* was comparable between the different samples, confirming the myocardial identity of all tissue samples that were collected (Fig.6B and C). Analysis of *HCN4* expression showed the highest relative expression in the SV-AVC continuity, with a lower level of expression in the AV canal and RV (Fig.6D). The difference in expression levels between the myocardial continuity and the RV was significant (*P* = 0.008). There was a trend towards significance in the difference between the continuity and the AV canal sample (*P* = 0.056). *ISL1* expression was only detectable in the samples taken from the SV-AVC continuity (Fig.6E).

Single cell patch-clamp was performed to functionally characterize the cells of the myocardial continuity between the sinus venosus and AV canal. Whole mount ISL1 staining was performed to identify the region of the continuity between the sinus venosus myocardium and posterior portion of the AV canal (Fig.6A). Cells from this region were compared to cells from the RV (Fig.6A). The action potential of cells from the SV-AVC continuity showed a pacemaker-like phenotype (Fig.6F), while the action potential of cells from the RV had a ventricular-like phenotype (Fig.6J). The SV-AVC cells had a higher spontaneous beating frequency (Fig.6H), a lower amplitude (Fig.6I), a shorter APD50% (Fig.6K) and APD90% (Fig.6L), in line with what is expected from pacemaker-like cells. The results are summarized in Table2.

**Table 2:** Electrophysiological characteristics

Electrophysiological parameter	SV-AVC (*n* = 8)	RV (*n* = 6)	*P*-value
V_max_ (V/s)	10.32 ± 4	9.45 ± 2	0.873
Frequency (Hz)	4.6 ± 0.3	2.6 ± 0.2	<0.001
APD50% (msec.)	52.73 ± 3	88.63 ± 11	0.004
APD90% (msec.)	72.79 ± 5	109 ± 11	0.007
Amplitude (mV)	82.66 ± 4.5	101.67 ± 2.8	0.006
MDP (mV)	−67 ± 2.7	−71.5 ± 1.3	0.209

APD: action potential duration; MDP: maximal diastolic potential; V_max_: maximal upstroke velocity; SV-AVC: cells from myocardial continuity sinus venosus and AV canal; RV: right ventricle.

Together these results confirm the pacemaker-like phenotype of the cells from the myocardial continuity between the sinus venosus and AV canal. Furthermore, results show that even during early development, it is possible to distinguish different cell populations within the region of the putative AVN.

### Physical lineage tracing shows incorporation of sinus venosus myocardium in the posterior region of the AV canal

The abovementioned results show the identification of an ISL1+/HCN4+/TNNI2+ myocardial continuity between the sinus venosus and posterior AV canal. At early developmental stages, ISL1-/TNNI2+ atrial myocardium is positioned between the ISL1+/TNNI2+ sinus venosus myocardium and the ISL1-/TNNI2+ AV canal myocardium. During further development, ISL1+/TNNI2+ myocardium is continuous with the ISL1-/TNNI2+ AV canal myocardium. To further study, the possible contribution from the ISL1+/TNNI2+ sinus venosus myocardium to the posterior portion of the AV canal, lineage tracing experiments were performed. *In ovo* microinjection experiments were performed in chicken embryos with vital dyes (DiI-5-TAMRA) aimed at labelling the ISL1+/TNNI2+ sinus venosus myocardium at stages when this myocardium is not yet in contact with the AV canal (HH14-15). This myocardial labelling was termed medial labelling, since the labelling was applied more medial than the second group of labelling performed in the mesenchyme outside the sinus venosus myocardium (termed lateral labelling). First, the results of the medial myocardial labelling will be discussed.

To confirm the location of the medial labelling, a group of embryos was harvested 1–2 hrs after microinjection (*n* = 14). DiI/5-TAMRA was found in the myocardium of the sinus venosus and the myocardium surrounding the RCV, both positive for ISL1 and TNNI2 (Fig.1A–C and E, 14/14 embryos, 100%; in Fig.1B all 14 separate microinjections are superimposed to clarify the extent of the total labelling). Labelling was also present in the mesenchyme surrounding the RCV (Fig.1D, 14/14 embryos, 100%). In 9/14 embryos (64%), a small area of labelling (on average 5.9% of total labelling) was also found in the ISL1-/TNNI2+ atrial myocardium directly bordering the ISL1+/TNNI2+ sinus venosus myocardium, but no leakage to the ISL1-/TNNI2+ connection between the inferior region of the atrium/sinus venosus and AV canal myocardium or the AV canal itself was seen. Limited leakage to other cardiac structures, such as the posterior OFT and ventricles was restricted to the upper layer of myocardial cells, while the labelling at the sinus venosus myocardium was applied to the full thickness of the myocardium. This first group of embryos therefore verified the correct location of injection. An interactive PDF of the three-dimensional reconstruction is available online, which shows the location of all separate initial microinjections (Data S1).

**Figure 7:**
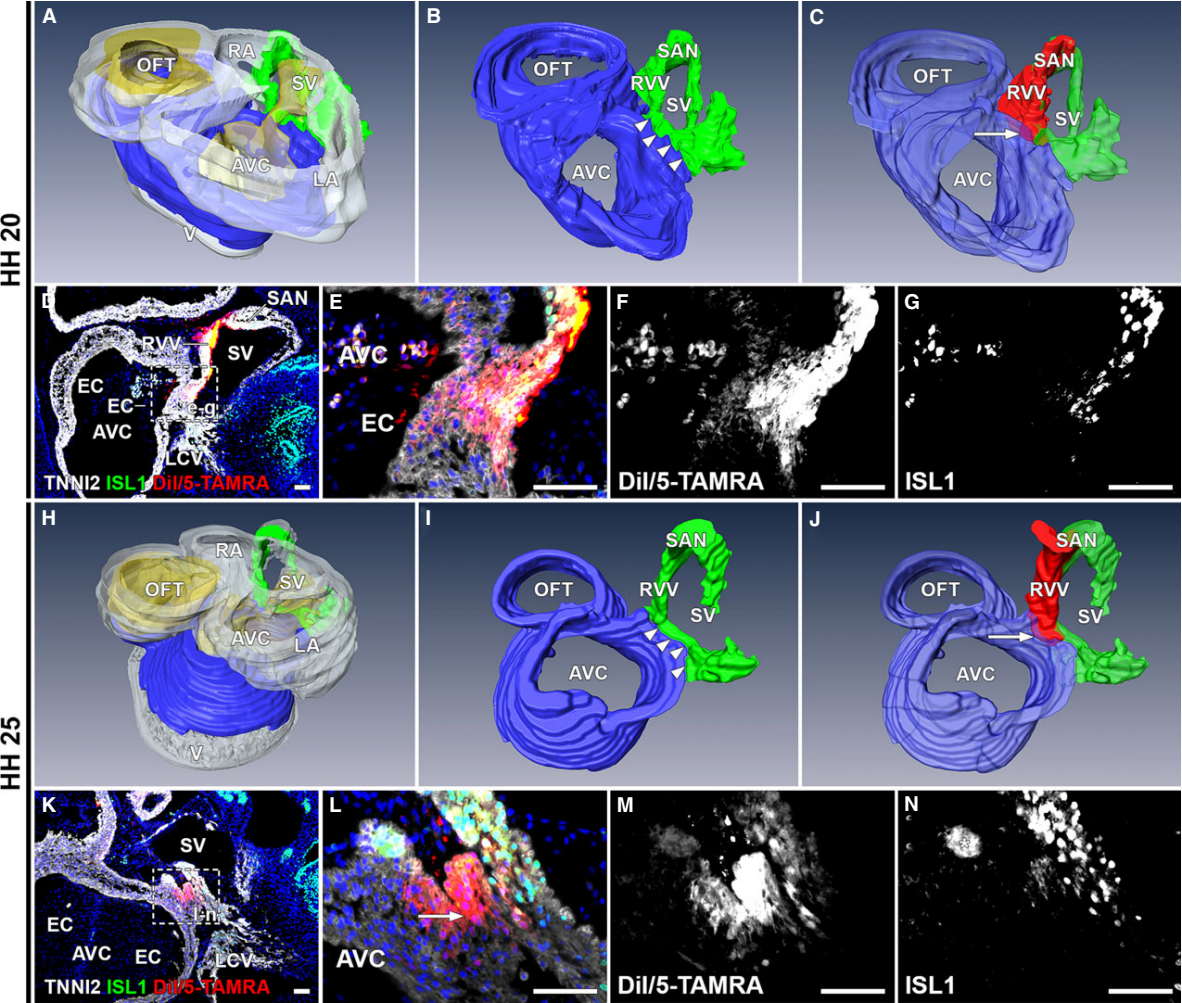
Tracing cells after medial labelling of ISL1+/TNNI2+ sinus venosus myocardium. (A–C) 3D reconstruction, HH20 embryo. White: myocardium, yellow: mesenchyme, blue: AV canal myocardium, green: ISL1+/TNNI2+ sinus venosus myocardium, red: DiI/5-TAMRA. (A) Overview of HH20 heart. (B) Detail of continuity (arrowheads) between AV canal and sinus venosus myocardium. (C) DiI/5-TAMRA labels SAN, RVV and right side myocardial continuity sinus venosus and posterior region of the AV canal (arrow). (D) Merge of DiI/5-TAMRA, ISL1, TNNI2 and DAPI. boxed area is shown at higher magnification in E–G. (E) Labelling (arrow) of continuity myocardium sinus venosus and AV canal. (F–G) Grey values of the fluorescent signal. (H) Overview of HH25 heart. (I) myocardial continuity at HH25 (arrowheads). (J) Labelling after 48 hrs is similar to 24 hrs and located in myocardial continuity sinus venosus and posterior part of the AV canal (arrow). (K) Overview of labelling at HH25. Magnification in L–N. (L) Shows DiI/5-TAMRA (arrow) in continuity between sinus venosus and AV canal myocardium. (M and N) Grey values of fluorescent signal. Asterisks in N: background staining. AVC: atrioventricular canal; EC: endocardial cushion; LCV: left cardinal vein; SV: sinus venosus. (D–G, K–N) Red: DiI/5-TAMRA, green: ISL1, white: TNNI2, blue: DAPI; scale bars: 50 μm.

Analysis of the embryos after 24 hrs of re-incubation (*n* = 10, HH19-22) revealed DiI/5-TAMRA in the sinus venosus myocardium (9/10 embryos, 90%), putative SAN (10/10 embryos, 100%), RVV (10/10 embryos, 100%), right atrium (7/10 embryos, 70%) and the continuity between the sinus venosus myocardium and the posterior AV canal myocardium (8/10 embryos, 80%; Fig.7A–G). The labelled area was largely positive for ISL1 and TNNI2 (Fig.7D–G). Two embryos did not show DiI/5-TAMRA in the continuity between the myocardium of the sinus venosus and AV canal. In one of these embryos, initial labelling was applied more cranially, only resulting in labelling of the atrial myocardium. The other embryo showed DiI/5-TAMRA in the caudal portion of the sinus venosus myocardium. Here, initial injection was applied more caudally than in the eight embryos that did show labelling of the continuity.

A third group of embryos was re-incubated for 48 hrs (*n* = 4, HH25). DiI/5-TAMRA was found in the myocardium of the putative SAN (3/4 embryos, 75%), the RVV (3/4 embryos, 75%), the sinus venosus myocardium (4/4 embryos, 100%), right atrium (3/4 embryos, 75%) and the myocardium of the posterior continuity between sinus venosus and AV canal (3/4 embryos, 75%; Fig.7H–N). In one embryo, labelling was not found in the continuity. This is explained by the more caudal initial injection that was performed, as was seen in one embryo in the second group.

### Lateral labelling of the RCV mesenchyme does not result in labelling of the myocardium of the sinus venosus or AV canal

The above-described results showed that in all embryos, DiI/5-TAMRA was found in the mesenchyme of the RCV. The labelled cells that were found in the myocardial continuity between sinus venosus and AV canal can therefore also be derived from this mesenchyme. To exclude this possibility, a second group of more lateral labelling of the RCV mesenchyme was performed at HH15-17 and the first group of embryos was harvested 1–2 hrs after injection (*n* = 6). Analysis showed DiI/5-TAMRA in the RCV mesenchyme and lateral body wall (6/6 embryos, 100%; Fig.1F–H). None of the analysed embryos showed leakage to myocardial structures.

**Figure 8:**
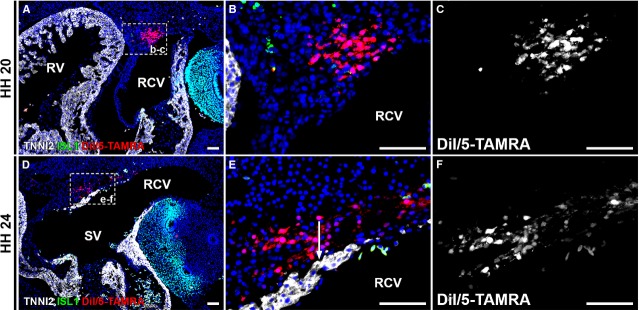
Tracing cells after lateral labelling of RCV mesenchyme. (A–C) After 24 hrs of re-incubation, labelling in wall of sinus venosus and RCV. (A) Overview, boxed area is shown in B and C. Labelling not present in myocardium and no ISL1+ cells labelled. (D–F) Re-incubation for 48 hrs shows comparable results, with labelling in mesenchyme of sinus venosus and RCV. No labelling in myocardium. Note sharp demarcation (arrow in E) between DiI/5-TAMRA labelled mesenchyme and TNNI2+ sinus venosus myocardium. No ISL1+ cells were labelled. RCV: right cardinal vein; RV: right ventricle; SV: sinus venosus. Red: DiI/5-TAMRA, green: ISL1, white: TNNI2, blue: DAPI; scale bars: 50 μm.

Analysis after 24 hrs of re-incubation (*n* = 5, HH19-21), showed labelling in the lateral body wall and TNNI2- RCV mesenchyme in all cases (5/5 embryos, 100%), with no labelling of myocardial structures, such as the myocardium of the RCV, RVV or sinus venosus (Fig.8A–C). After 48 hrs of re-incubation (*n* = 5, HH24-25), DiI/5-TAMRA was found in the TNNI2- mesenchyme of the RCV (5/5 embryos, 100%; Fig.8D–F). None of the embryos showed labelling of the SAN, RVV or sinus venosus myocardium.

Together, the medial and lateral labelling experiments show that ISL1+/TNNI2+ myocardium of the sinus venosus is incorporated in the posterior region of the AV canal.

## Discussion

The adult AVN consists of multiple cell types (*i.e*. compact nodal cells, transitional cells and nodal extensions) with different morphological and electrophysiological characteristics. The initiation of AVNRT requires the presence of pathways with different electrophysiological characteristics, resulting in slow conduction in the so-called slow pathway, unidirectional conduction in the fast pathway and a central area of block. Cellular and functional heterogeneity of the node is thus a prerequisite for AVNRT to occur. A key finding of this study was the demonstration of incorporation of sinus venosus myocardium in the AV canal in the posterior region of the AVN.

It has been suggested that the transitional cells of the AVN are an atrial contribution to the AVN 25. The inferior nodal extension, which acts as the slow pathway in AVNRT, has been described as atrial myocardium, based on histological criteria 16. The CCS-LacZ 26,27 and HCN4nLacZ 28 mice, which were used to visualize the developing CCS, demonstrated that the sinus venosus myocardium – that includes the SAN at its proximal end as well as the left and right venous valves – is connected to the AVN. Analysis of protein expression of HNK-1 in human embryos 29, mRNA expression of *HCN4* in chicken embryos 23 and *Tbx3* in mice 30 showed the same connection between the sinus venosus myocardium and the AVN. More recently, it was established that Isl1+ cardiogenic mesoderm contributed to the AVN 20. These results suggest that the sinus venosus myocardium and venous valves are connected with the AVN through specialized myocardium of the CCS. However, these studies did not show a direct embryonic contribution from the sinus venosus myocardium to the developing AV canal. To overcome these limitations, it is necessary to directly label the cells from the sinus venosus myocardium and trace these cells during development.

Recently, two separate research groups performed vital dye labelling experiments and showed a very early contribution (4–6 somite stage in mouse 31 and HH8 in chick 32) from a region posterior to the cardiac crescent to the AV canal myocardium. Domínguez *et al*. showed that the majority of cells comprising the murine AV canal are derived from an *Isl1*+ and *Mlc2a*- pool of cells 31. Bressan *et al*. showed a contribution to the developing AV canal from an even more posteriorly located region, which is *Isl1*- and *Nkx2-5*- 32. These experiments suggest that the AV canal myocardium, and thus the AVN, is derived from a larger population of cells than cardiac crescent cells alone. However, these results still do not prove the addition of cells from the sinus venosus myocardium to the developing AV canal.

This study is aimed at the area of myocardial continuity between the sinus venosus and AV canal. Expression data show that in this tissue, ISL1 is strongly expressed in the sinus venosus myocardium and decreases towards the AV canal. NKX2-5 expression shows a similar but opposite pattern, with an increase in expression towards the AV canal myocardium, suggesting that ISL1+ cells are differentiating towards a more restricted cardiac fate in this region. The continuity between sinus venosus myocardium and the AV canal also showed *HCN4* mRNA expression and was identified in murine embryos co-expressing ISL1, TNNI2 and HCN4. HCN4 is responsible for the ‘funny current’, enabling cardiomyocytes to depolarize spontaneously and HCN4 is known to be expressed in the (developing) CCS 23. The pacemaker-like phenotype of the myocardial continuity was confirmed with single cell patch-clamp experiments and by qPCR. The different phenotype of this region compared to the more caudal, posterior region of the AV canal was demonstrated by the different levels of mRNA expression of *HCN4* and *ISL1*. These results suggest the presence of distinct, heterogenic cell populations within the region of the putative AVN and indicate a contribution of the sinus venosus during the development of the AVN.

Vital dye labelling of the ISL1+/TNNI2+ sinus venosus myocardium at HH15 (when this myocardium was not yet in contact with the AV canal) and follow-up of these cells during further development, showed that part of the sinus venosus myocardium becomes incorporated in the posterior region of the AV canal myocardium (putative AVN region). As mentioned, it was shown that in the region of the developing AVN, different cell populations (*i.e*. relative high expression of *HCN4* and *ISL1* in the myocardial continuity between the sinus venosus and AV canal as compared to the more caudal portion of the posterior AV canal) can be distinguished, even early in development.

These results combined with the co-expression studies support the hypothesis that the definitive AVN is derived from different sources of cells that are added at different time-points. We hypothesize a relatively late (after HH15, when the PHT has been formed and dextral looping has progressed) contribution from the sinus venosus myocardium to the developing AVN, which results in the presence of multiple cell types in the adult AVN. Furthermore, we postulate that the cells from this sinus venosus-AV canal continuity contribute to the nodal extensions or transitional cells of the AVN, as the continuity is located posterosuperiorly in the AV canal.

Using a genetic lineage tracing approach in mice, Aanhaanen *et al*. described no contribution of Tbx18+ (used as a marker for the sinus horns myocardium and the epicardium) cells to the AVN or AV junction 13. The current study describes a contribution from the sinus venosus myocardium to the AV canal. A possible explanation for this difference could be the different markers used to identify the sinus venosus myocardium. The Tbx18-cre used will probably label a different pool of cells as compared to the ISL1+ cells that were labelled in this study. Furthermore, it has been shown that inefficient recombination of the ROSA26R locus 33 (the reporter that was used in the Tbx18-Cre based genetic tracing experiments mentioned above was the R26R-LacZ mouse) makes it difficult to interpret the results. Ma *et al*. reassessed the fate of Isl1+ and Nkx2-5+ cells and compared the results of a ROSA26R-based or GATA4-based reporter system. They found large differences in recombination domains between the two reporter systems, with a far larger recombination domain for the GATA4-based reporter experiments 33. These results show that it is difficult to draw conclusions from the absence of reporter gene expression.

Two limitations of the vital dye experiments are the potential leakage of dye to adjacent structures and the impossibility to directly correlate the initial labelling to the fate of cells at a later time-point within the same embryo. Leakage to the ISL1-/TNNI2+ atrial myocardium was calculated and showed that only a small percentage (on average 5.9%) of labelling was found in this region. Furthermore, labelling experiments in a large number of embryos and the high reproducibility of injections and pattern of distribution minimized these limitations.

To describe the dynamics of ISL1 expression, early stages of development were analysed and co-expression of ISL1 and TNNI2 was found in the bilateral cardiogenic plates, before formation of the PHT. Cells that contribute to the cardiac crescent and PHT are considered to be derivatives of the first heart field. Mesodermal cells at both poles of the tube which differentiate and are recruited to the heart are considered to be derivatives of the second heart field 34. *Isl1* is commonly used as a marker for the second heart field. However, co-expression of ISL1 and TNNI2 was found in the majority of cells of the bilateral cardiogenic plates (first heart field derived) in chicks, which is in agreement with expression of *ISL1* mRNA in this region 35. As described above, Ma *et al*. showed that the majority of cardiac cells (including LV cardiomyocytes) are derived from *Isl1*+ progenitors 33. This shows that *Isl1* is not suitable as a second heart field marker 33. These results raise the question whether the first and second heart field should be considered as truly distinct cell populations, or rather form a continuum and reflect different stages of differentiation of progenitor cells with a cardiac fate, as was mentioned previously 33,36. Nevertheless, the analysis of ISL1 expression, which was performed in this study, is crucial to identify cardiac progenitors and investigate the differentiation state of (pre)cardiac cells.

This study describes co-expression of ISL1 and TNNI2 beyond HH12 in regions relevant for normal CCS development, but also in myocardial structures known to underlie common arrhythmias. Furthermore, it was shown that the myocardial continuity in the upper region of the developing AVN shows relative high expression of *HCN4* and *ISL1*. Our data and the results from other studies point towards an important link between Isl1 and the CCS. The primary pacemaker of the heart, the SAN, retains ISL1 expression until late stages of development, whereas in the remaining heart, ISL1 expression is down-regulated after cells differentiate to working cardiomyocytes 2. Previous inducible Cre-based genetic tracing experiments showed the contribution of Isl1+ progenitors to the SAN and AVN 20. Also, a link between Shox2, a transcription factor essential in regulating the SAN gene expression program, and Isl1 has been shown 37. Mice lacking Shox2 present with severe bradycardia and defects in SAN development, and show down-regulation of Isl1 expression in the SAN. The Shox2-mediated bradycardia could be rescued by Isl1 in a zebrafish model 37. Zebrafish lacking isl1 expression show pacemaker dysfunction and the isl1+ cells from the inflow portion of the heart reveal pacemaker activity 38 A possible mechanism by which ISL1 is involved in CCS functioning is by keeping cells in a primitive state, maintaining their pacemaker-like phenotype and preventing them from further differentiating towards working myocardium. The CCS consists of cells resembling a more primitive myocardium with slow conduction, high automaticity, and poor electrophysiological coupling 10. We hypothesize that selected clinical arrhythmias could be explained by a local diminished or absent down-regulation or re-expression of Isl1 resulting in areas with a more primitive, ‘CCS-like’ phenotype, from which arrhythmias originate. Further experiments are needed to test this hypothesis.

### Conclusion

Incorporation of sinus venosus myocardium in the posterior part of the AV canal myocardium was demonstrated. It is postulated that the sinus venosus myocardium contributes to the nodal extensions or transitional cells of the AVN. Multiple sources of cells contributing to the AVN may explain the heterogeneity of the node, and form the substrate underlying AV nodal reentrant tachycardia.

## Supporting Information

**Data S1** 3D interactive PDF, HH15.

**Data S2** 3D interactive PDF, HH21.

**Figure S1** Representative images of regions collected by LCM.
