# Supplementary material for: The sinus venosus myocardium contributes to the atrioventricular canal: potential role during atrioventricular node development?
Source: J Cell Mol Med. 2015 Mar 6;19(6):1375–89. doi: 10.1111/jcmm.12525 (PMC4459851; doi:10.1111/jcmm.12525)
Supplement: Supplementary file 1 [file jcmm0019-1375-sd1.pdf]

Legend online interactive 3D PDF S1, HH15 heart

Required settings:
In order to correctly use the interactive PDF, use the settings below, adapted and slightly modified after De Boer *et al* (de Boer et al., 2011)

- Use Adobe Acrobot® version 9.3 or higher
- Under “Edit” → “Preferences” → 3D & Multimedia → 3D Tool Options
    - “Open Model Tree on 3D activation” choose “No”
    - “Default Toolbar State” choose “Hidden”
    - Disable “Show 3D Orientation Axis”
- Under “Edit” → “Preferences” → 3D & Multimedia → Auto-Degrade Options
    - “Optimalization Scheme for Low Framerate” choose “None”
- Under “Edit” → “Preferences” → Javascript
    - Make sure “Enable Acrobat Javascript” is switched on

How to use the 3D PDF
Activate: click on reconstruction
Move: hold the left mouse button and move the mouse
Zoom: scroll up and down with the mouse wheel or hold the right mouse button and move up and down with the mouse
Pre-programmed views: on the right side of the reconstruction under “Views”, four pre-programmed views are available, which are activated by clicking on the small thumbnail of the view
Show, hide or make structures transparent: The different structures (e.g. the AV canal myocardium) that make up the reconstruction can be shown, hidden or made transparent by clicking on the “+” (=show), “+/-” (=transparent) or “-” (=hide) buttons underneath the desired structure (see below). In this way, the 14 separate labels can be made visible, transparent or invisible (e.g. for embryo 1, click E1+ (=show), E+/- (=transparent) or E- (=invisible), see below for E1).

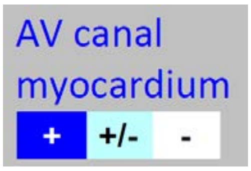

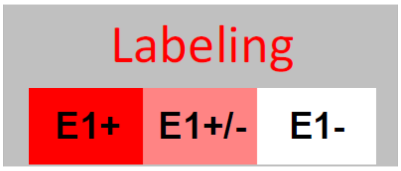

Description of the reconstruction
In this reconstruction, a HH15 heart is reconstructed. This reconstruction shows 1. the ISL1-/TNNI2+ myocardium between the ISL1+/TNNI2+ sinus venosus myocardium (green) and the ISL1-/TNNI2+ AV canal myocardium at this stage and 2. the individual location of all 14 embryos that were labeled and analysed after 1-2 hours of incubation (initial medial labelling). The first view (upper-left button) shows a frontal view of the heart. The second view (upper-right button) shows the left lateral view, clearly showing the white ISL1-/TNNI2+ myocardium between the sinus venosus myocardium (green) and AV canal myocardium (blue). This is also displayed in the third view (lower-left button), but from the right dorsolateral side. In this view, all important structures are annotated. The following abbreviations are used: AV: atrioventricular; LCV: left cardinal vein; OFT: outflow tract; RCV: right cardinal vein; SV: sinus venosus. The DiI/5-TAMRA labeled region (red) is also clearly visible. All labels can be made transparent or invisible by clicking the corresponding buttons on the right of the screen, under “Labeling”. The fourth view (lower-left button) shows the distance between the ISL1+/TNNI2+ myocardium (green) and the AV canal myocardium (blue), with all 14 labels transparent.

Reference
De Boer BA, Soufan AT, Hagoort J, Mohun TJ, van den Hoff MJB, Hasman A, Voorbraak FPJM, Moorman AFM, Ruijter JM. 2011. The interactive presentation of 3D information obtained from reconstructed datasets and 3D placement of single histological sections with the 3D portable document format. Development 138:159–167.

3D reconstruction HH15 heart + vital dye labeling location

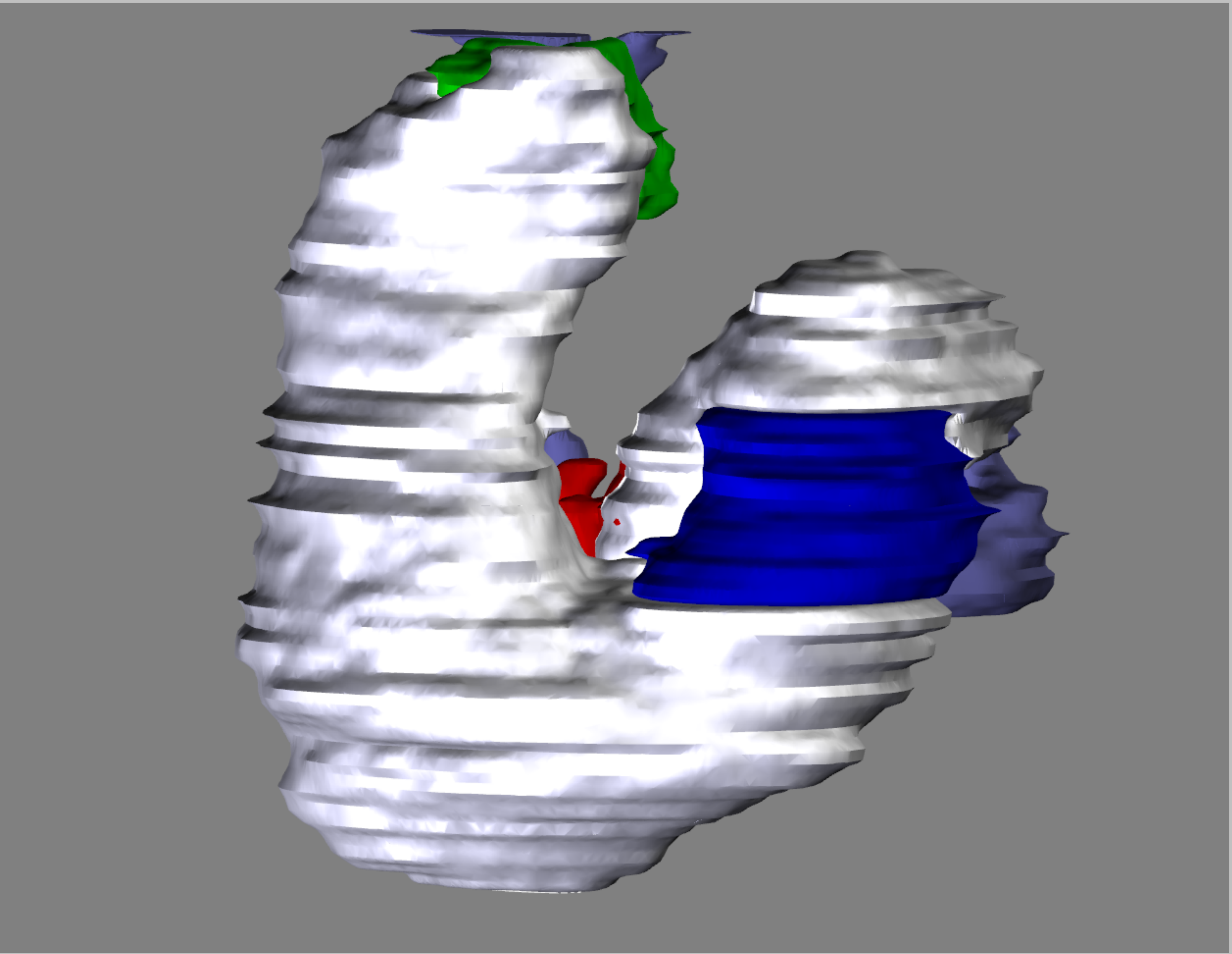

Views

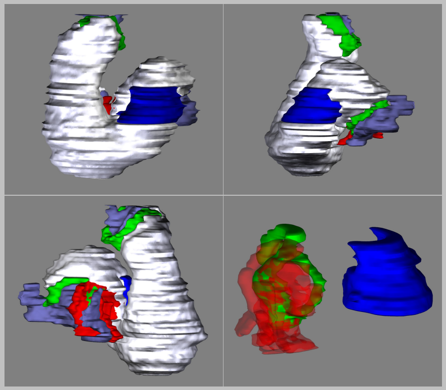

Labeling

E1+	E1+/-	E1-
E2+	E2+/-	E2-
E3+	E3+/-	E3-
E4+	E4+/-	E4-
E5+	E5+/-	E5-
E6+	E6+/-	E6-
E7+	E7+/-	E7-
E8+	E8+/-	E8-
E9+	E9+/-	E9-
E10+	E10+/-	E10-
E11+	E11+/-	E11-
E12+	E12+/-	E12-
E13+	E13+/-	E13-
E14+	E14+/-	E14-

AV canal myocardium

+	+/-	-

ISL1+/TNNI2+ myocardium

+	+/-	-

Myocardium

+	+/-	-

Lumen

+	+/-	-
