# Supplementary material for: The sinus venosus myocardium contributes to the atrioventricular canal: potential role during atrioventricular node development?
Source: J Cell Mol Med. 2015 Mar 6;19(6):1375–89. doi: 10.1111/jcmm.12525 (PMC4459851; doi:10.1111/jcmm.12525)

Legend online interactive 3D PDF S2, HH21 heart

Required settings:
In order to correctly use the interactive PDF, use the settings below, adapted and slightly modified after de Boer *et al* (de Boer et al., 2011)

- Use Adobe Acrobot® version 9.3 or higher
- Under “Edit” → “Preferences” → 3D & Multimedia → 3D Tool Options
    - “Open Model Tree on 3D activation” choose “No”
    - “Default Toolbar State” choose “Hidden”
    - Disable “Show 3D Orientation Axis”
- Under “Edit” → “Preferences” → 3D & Multimedia → Auto-Degrade Options
    - “Optimalization Scheme for Low Framerate” choose “None”
- Under “Edit” → “Preferences” → Javascript
    - Make sure “Enable Acrobat Javascript” is switched on

How to use the 3D PDF
Activate: click on reconstruction
Move: hold the left mouse button and move the mouse
Zoom: scroll up and down with the mouse wheel or hold the right mouse button and move up and down with the mouse
Pre-programmed views: on the right side of the reconstruction under “Views”, three pre-programmed views are available, which are activated by clicking on the small thumbnail of the view
Show, hide or make structures transparent: The different structures (e.g. the AV canal myocardium) that make up the reconstruction can be shown, hidden or made transparent by clicking on the “+” (=show), “+/-” (=transparent) or “-” (=hide) buttons underneath the desired structure (see below).

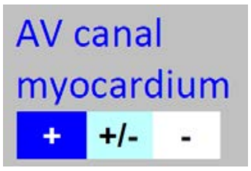

Description of the myocardial continuity between the sinus venosus and posterior AV canal
This reconstruction clarifies the three-dimensional relation of the continuity between the ISL1+/TNNI2+ sinus venosus myocardium (green) and the AV canal myocardium (blue). On the right, under “Views”, three pre-programmed views are available. The first view gives a frontal view of the heart at HH21. The second view shows the three-dimensional location of the AV canal (blue) and ISL1+/TNNI2+ sinus venosus myocardium (green), in relation to the rest of the heart and mesenchymal tissue. The third view shows the myocardial continuity between the sinus venosus and posterior AV canal.

Reference
De Boer BA, Soufan AT, Hagoort J, Mohun TJ, van den Hoff MJB, Hasman A, Voorbraak FPJM, Moorman AFM, Ruijter JM. 2011. The interactive presentation of 3D information obtained from reconstructed datasets and 3D placement of single histological sections with the 3D portable document format. Development 138:159–167.

3D reconstruction HH21 heart

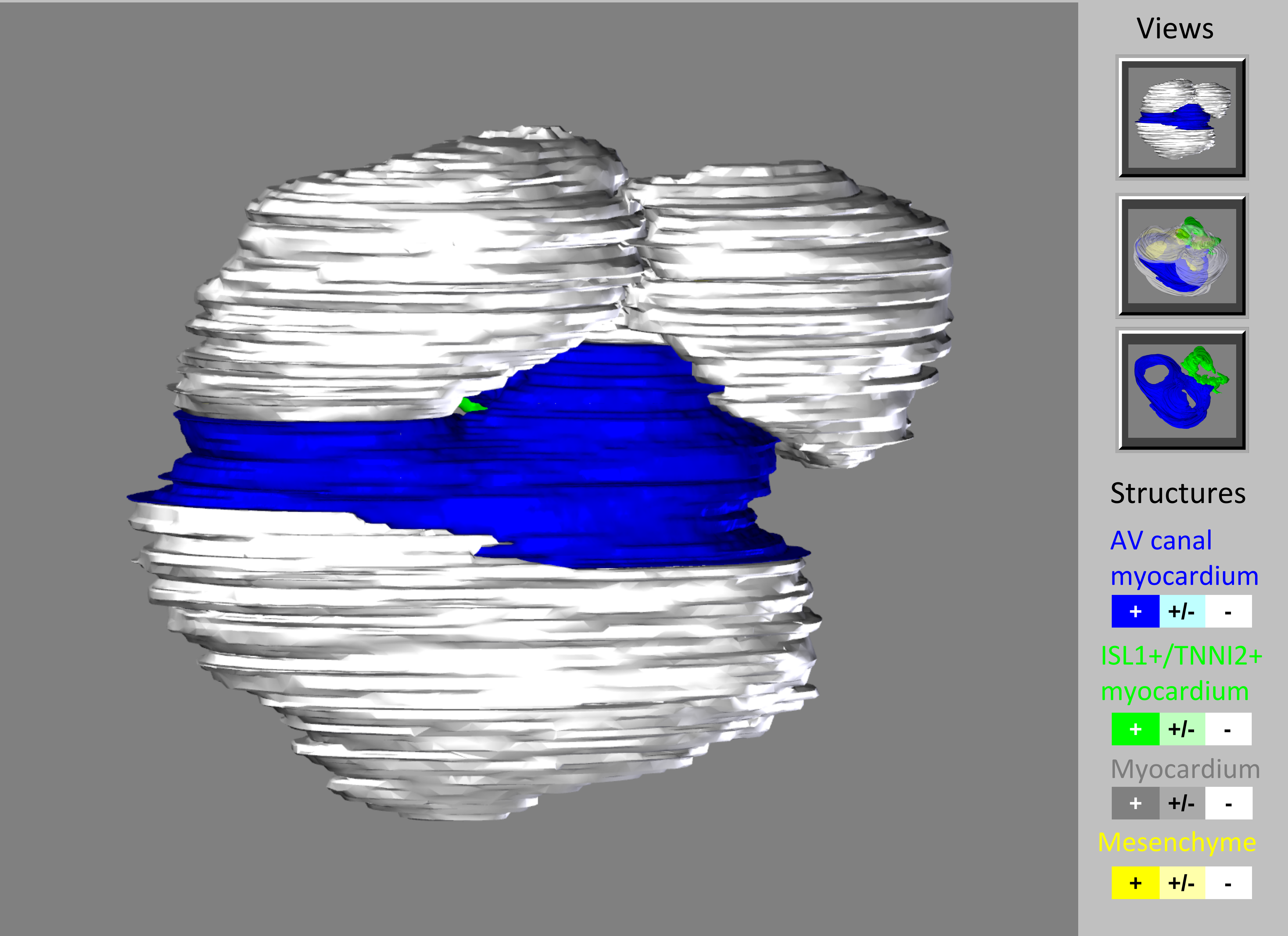

Supplement: Supplementary file 2 [file jcmm0019-1375-sd2.pdf]
