# Supplementary figures and images for: The sinus venosus myocardium contributes to the atrioventricular canal: potential role during atrioventricular node development?
Source: J Cell Mol Med. 2015 Mar 6;19(6):1375–89. doi: 10.1111/jcmm.12525 (PMC4459851; doi:10.1111/jcmm.12525)

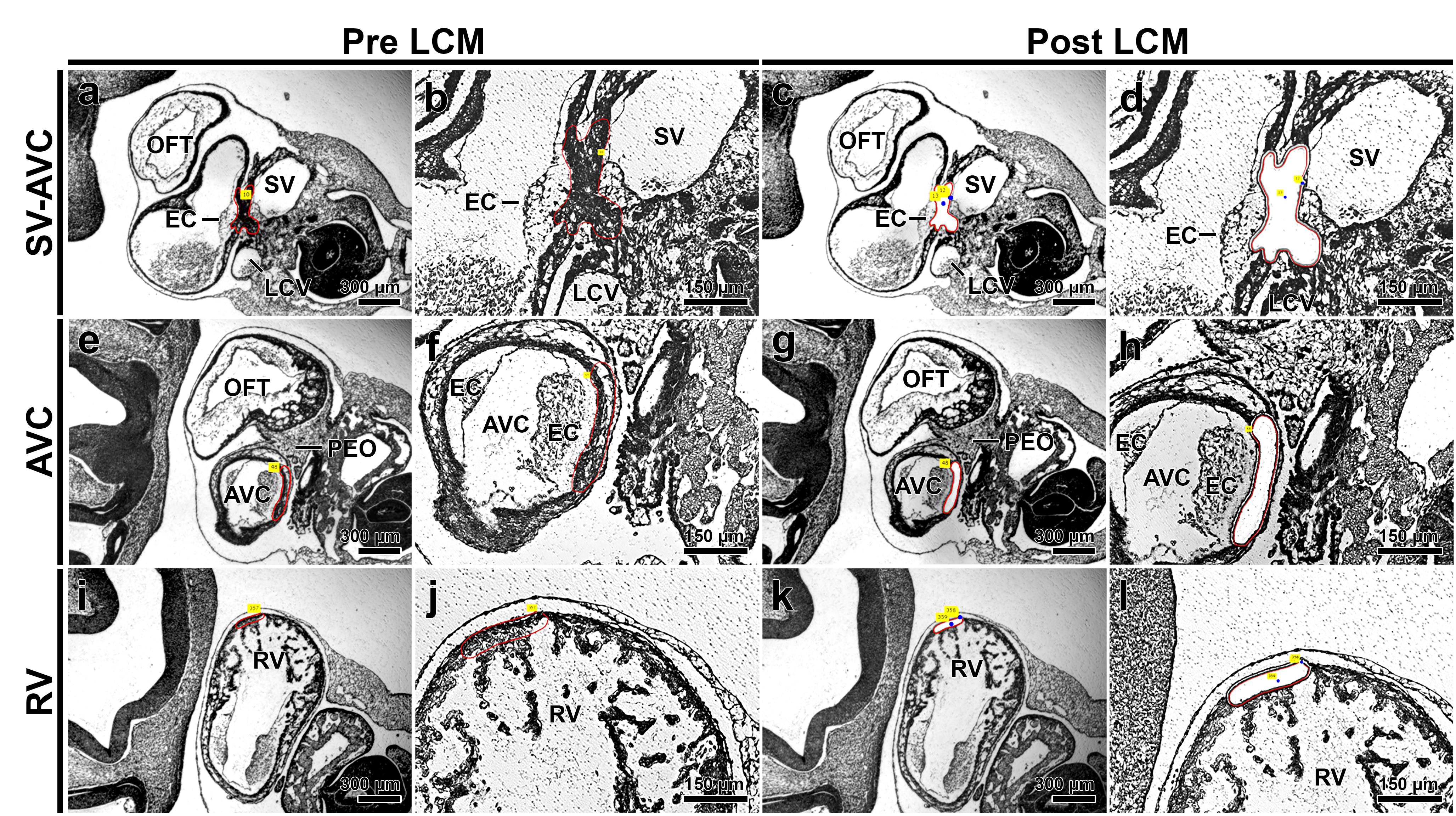

Supplement: Supplementary file 3 [file jcmm0019-1375-sd3.tif]
